# The Book World of Medicine and Science

**Published:** 1897-06-05

**Authors:** 


### The Book World of Medicine and Science.

The Climate of Bournemouth in Relation to Disease. By A. Kinsey Morgan, M.D. 8vo., pp. 51. (Bristol: John Wright and Co. 1897.)

Too often writers on the virtues of health resorts damn, not with faint but with fulsome praise, the claims of various places to recognition as valuable therapeutical resources, and we therefore feel that Dr. Kinsey Morgan's moderate language in setting before us the facts in favour of Bournemouth as a place of residence for phthisical, bronchitic, and over-worked patients only adds weight to his thesis. We have long held that the more favoured spots in England should offer climatic advantages to many patients which cannot be secured elsewhere; and seeing that in addition the difficulties and expense of reaching far off countries are much greater, there can be little doubt that Bournemouth will continue to grow in favour. The little brochure before us affords evidence of scientific culture, and will be read with advantage by every practitioner who has cases on his hands for whom a healthy environment and change of air has become essential for recovery.

Sanitation and Health. By Brig.-General R. C. Hart, V.C., C.B., R.E. Revised by Brig.-Surg. Lieut.-Col. T. H. Hendley, C.I.E., M.R.C.S., L.R.C.P., &c. Authorised by the War Office for use in all Army Schools. Third edition. (London: William Clowes and Sons, Limited, 13, Charing Cross. 1897. Price 1s. 6d. 37 pages.)

The appearance of a third edition of this little book shows that it remains a popular and useful one, its very slightness helping to make it comprehensible to every adult human being who is able to read it. There is, perhaps, a certain monotony about it due to the absence of emphasis on some of the more important matters of which it treats. For instance, "avoid stimulants" is advice given to the soldier in India for many reasons, but it hardly puts strongly enough the surpassingly active part taken by even a moderate use of stimulants in the production of sunstroke. Again, properly trapped drains are always of importance where they can be had, but there are other points in reference to house drains which deserve explanation over and above casual mention. The book is, however, a readable one, and will probably give many a useful hint to those who, however desirous of leading a healthy life, are unable to study the subject in any more elaborate work.

